# Integrative Multi‐Omics‐Informed Analysis Identifies Insulin Therapy–Associated Loci Implicated in Osteoarthritis Susceptibility

**DOI:** 10.1111/cbdd.70384

**Published:** 2026-08-26

**Authors:** Lingbo Rong, Jiayong Zhu, Yifan Liu, Tianyu Dai, Liaobin Chen, Hui Wang

**Affiliations:** ^1^ Department of Pharmacology Wuhan University School of Basic Medical Sciences Wuhan China; ^2^ Department of Orthopedic Surgery Zhongnan Hospital of Wuhan University Wuhan China; ^3^ Department of Pediatric Surgery Zhongnan Hospital of Wuhan University Wuhan China; ^4^ Hubei Provincial Key Laboratory of Developmentally Originated Disease Wuhan China

**Keywords:** cartilage matrix degradation, exogenous insulin, functional annotation, genetic variants, immune inflammation, mendelian randomization, metabolic regulation, NRF2, osteoarthritis

## Abstract

Integrative genetic approaches can combine Mendelian randomization, variant annotation, pathway‐level interpretation, and exploratory cellular measurements to investigate complex disease mechanisms. However, the relationship between genetic liability to insulin medication use and osteoarthritis (OA) remains incompletely characterized GWAS summary statistics for insulin medication use were obtained from the UK Biobank insulin medication‐use dataset. Insulin therapy‐associated SNPs were selected using a suggestive significance threshold (*p* < 5 × 10^−6^) and clumped (*r* ^2^ < 0.001, 10,000 kb). Two‐sample Mendelian randomization (MR) was performed using the inverse‐variance weighted (IVW) method as the primary analysis, supplemented by MR‐Egger, weighted median, simple mode, and weighted mode methods. Sensitivity analyses included Cochran's *Q* test, MR‐Egger intercept, MR‐PRESSO global test, and leave‐one‐out analysis. Insulin‐associated genetic variants were functionally annotated using Ensembl VEP, ANNOVAR, FUMA, and Open Targets Genetics, and mapped genes were subjected to exploratory GO biological process and KEGG pathway enrichment analysis. A separate exploratory cellular experiment was performed in the immortalized human chondrocyte cell line TC28a2 using insulin treatment followed by Safranin O staining, RT‐qPCR, western blotting, and ELISA. After SNP selection and harmonization, 56 instrument‐outcome associations were retained, comprising 29 SNPs in the ukb‐a‐106 OA dataset and 27 SNPs in the ukb‐b‐14,486 OA dataset. IVW analysis suggested an association between genetic liability to insulin medication use and increased OA susceptibility (ukb‐a‐106: OR = 1.195, 95% CI = 1.062–1.344, *p* = 0.003; ukb‐b‐14,486: OR = 1.179, 95% CI = 1.066–1.305, *p* = 0.001). Sensitivity tests did not detect statistically significant heterogeneity or directional horizontal pleiotropy; however, indication‐related genetic liability, correlated pleiotropy, and biologically plausible alternative pathways remain important concerns. Seven mapped genes were used for exploratory enrichment analysis. In a separate exploratory cellular experiment, insulin‐treated TC28a2 cells under high‐glucose conditions showed reduced proteoglycan staining, increased matrix‐degrading and inflammatory markers, and reduced NRF2 protein expression. This study suggests an association between genetic liability to insulin medication use and OA susceptibility and provides exploratory functional‐annotation and cellular observations for further investigation.

## Graphical abstract

Integrative multi‐omics‐informed analysis links insulin therapy–associated germline variants to increased osteoarthritis susceptibility. Genomic annotation, pathway enrichment, and transcript/protein‐level chondrocyte validation implicate metabolic dysregulation, immune‐inflammatory activation, NRF2 suppression, and cartilage matrix degradation as mechanisms underlying insulin‐related joint damage.

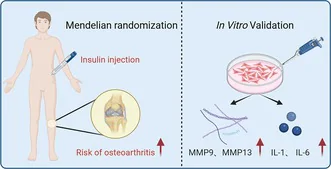

## 1 Introduction

Osteoarthritis (OA) is a chronic degenerative joint disease characterized by the breakdown of cartilage and underlying bone (Abramoff and Caldera 2020). Patients with OA typically experience joint pain, stiffness, and reduced mobility, which significantly impact their daily activities. The condition is often accompanied by subchondral bone sclerosis, synovial inflammation, and periarticular soft tissue changes, which can lead to severe disability. The prevalence of OA is increasing due to the aging population and the rising prevalence of obesity, posing a significant public health challenge in the coming decades (Barnett 2018; Askari et al. 2017). Treatments for OA today mainly include using drugs and artificial joint replacement; however, these treatments are focused on improving the symptoms of joint pain rather than halting the progression of the disease (Xia et al. 2014). In recent years, there has been a growing emphasis on early prevention strategies to halt or delay the progression of OA before substantial cartilage damage occurs.

Diabetes is a chronic metabolic disease characterized by defective insulin secretion and/or impaired insulin action, leading to elevated blood sugar levels compared to healthy individuals and posing a significant threat to human health (Lewis and Brubaker 2021). Interestingly, it has been proposed that several shared factors may contribute to the development of both OA and diabetes. While insulin is one of the most commonly used medications in diabetic patients, the impact of exogenous insulin therapy on OA remains a subject of debate (Bowden et al. 2019; Veronese et al. 2019). Traditional observational studies often face challenges such as confounding bias and reverse causality, which limit their ability to establish a clear cause‐and‐effect relationship. Although randomized controlled trials (RCTs) are considered the gold standard for determining causality (Bhide et al. 2018), they can be limited by substantial economic and temporal constraints. In some cases, the assignment of exposures may also be deemed impractical or unethical (Nielsen et al. 2020; Allen et al. 2015; Mani and Björck 2019). Despite these limitations, the relationship between exogenous insulin and OA remains unclear, highlighting the need for further investigation.

Mendelian randomization (MR) is an analytical approach that uses genetic variants as instrumental variables to infer the potential causal effect of an exposure on an outcome by leveraging the random assortment of alleles at conception, thereby minimizing confounding and reverse causation biases commonly encountered in observational studies (Birney 2022; Bowden et al. 2015). In recent years, MR has been widely applied to explore causal relationships between lifestyle factors and OA, becoming a powerful tool for drug target discovery and validation (Bowden and Holmes 2019; Sekula et al. 2016; Alhassan et al. 2024).

Beyond MR estimation, functional annotation and gene‐level analyses may improve the biological interpretation of inherited variants. However, variant annotation, pathway enrichment, and targeted mRNA or protein measurements should not be described as transcriptomic or proteomic profiling when no omics‐scale datasets are generated.

In this study, we performed a two‐sample MR analysis to evaluate the association between genetic liability to insulin medication use and OA risk. We then conducted functional annotation and exploratory GO/KEGG analyses to identify biological themes linked to the selected variants. Finally, a separate exploratory experiment in immortalized human chondrocytes examined short‐term cellular responses to insulin, including cartilage matrix‐related, inflammatory, and NRF2‐associated measurements. The genetic and cellular components address different exposure constructs and are therefore interpreted as complementary but non‐validating lines of investigation.

## 2 Materials and Methods

### 2.1 Experimental Design

**FIGURE 1:**
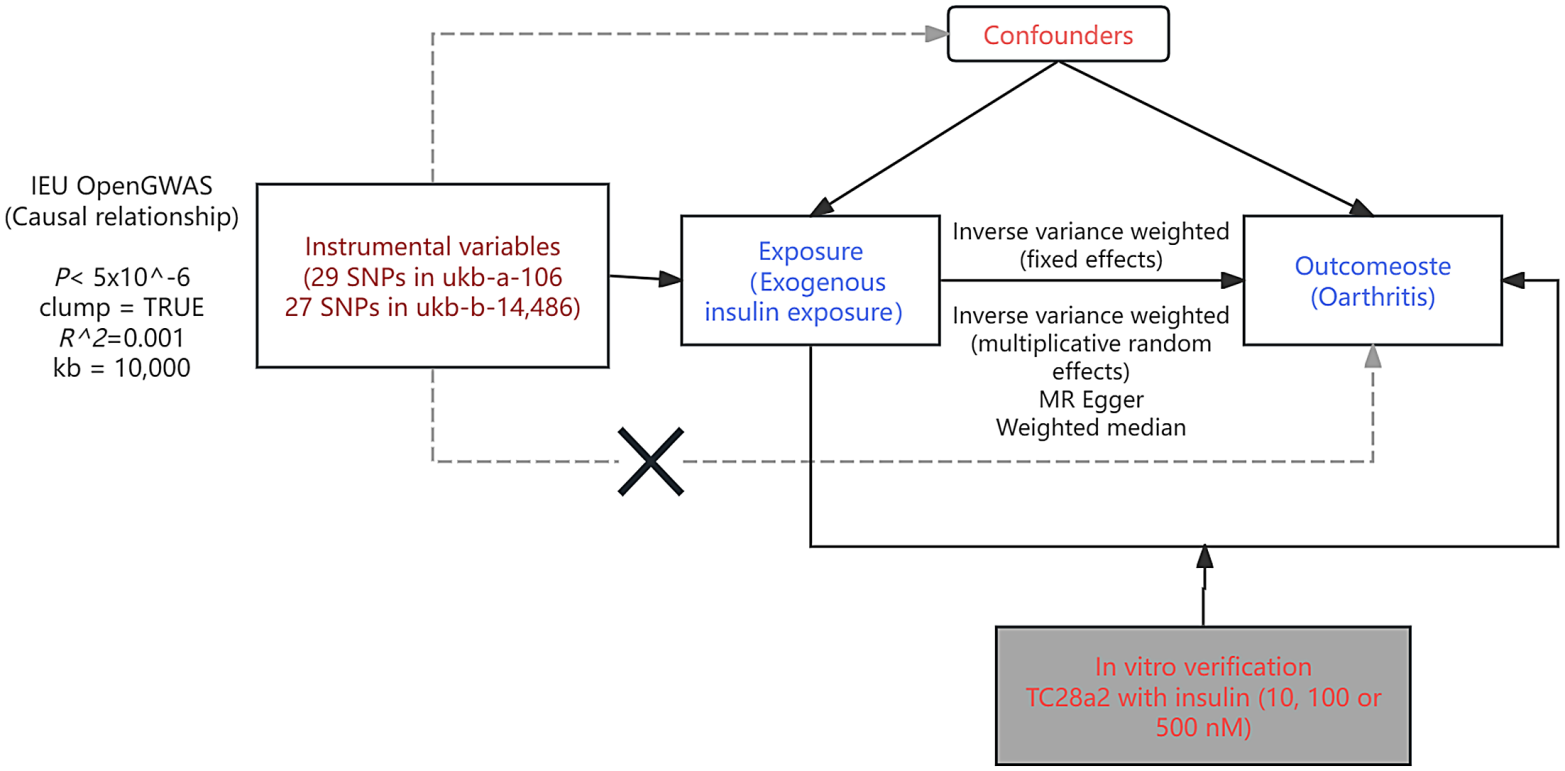
Study design integrating two‐sample Mendelian randomization, functional annotation, exploratory enrichment analysis, and a separate exploratory cellular experiment.

We applied two‐sample Mendelian randomization using SNPs as instrumental variables to evaluate the association between genetic liability to insulin medication use and OA. The selected instruments were expected to satisfy three assumptions: relevance to the exposure, independence from confounders, and absence of pathways to OA other than through the exposure. Because the exposure is a medication‐use phenotype that overlaps genetically with diabetes liability and treatment indication, the second and third assumptions cannot be considered fully guaranteed. The separate cellular experiment was exploratory and was not designed as validation of the MR results. The overall study design is shown in Figure 1. Because the genetic analyses used publicly available summary data (Burgess et al. 2015), no additional ethical approval was required. Detailed procedures for instrument evaluation, cell culture, and statistical analysis are provided in the Materials and Methods—Supporting Information S1.

### 2.2 Data Sources and Pre‐Processing

The genetic instruments for insulin medication use were derived from the UK Biobank GWAS dataset ukb‐a‐153, which captures self‐reported use of insulin products based on treatment/medication codes (UK Biobank data category: “Treatment/medication code: insulin product”). Participants were classified as exposed if they had ever been prescribed or self‐reported insulin‐containing medications (e.g., human insulin, insulin analogues, or combination products containing insulin), whereas non‐exposed participants had no record of insulin medication use. Thus, the exposure should be interpreted as an insulin medication‐use phenotype, not as endogenous insulin secretion, pancreatic beta‐cell function, diabetes status, or a randomized insulin‐treatment contrast. Because insulin use is strongly determined by diabetes diagnosis, disease severity, and treatment indication and may share genetic architecture with diabetes‐related traits, this exposure raises concerns regarding indication‐related genetic liability, correlated pleiotropy, and potential horizontal pleiotropy. These concerns are explicitly considered in the Discussion and Limitations.

The Integrated Epidemiology Unit Open GWAS database was used to obtain summary statistics (Alhassan et al. 2024). Genetic instruments for insulin medication use were derived from ukb‐a‐153, and OA outcomes were obtained from ukb‐a‐106 and ukb‐b‐14,486 (Wang et al. 2023) (Table S2). A suggestive threshold of *p* < 5 × 10^−6^ followed by clumping (*r* ^2^ < 0.001; 10,000 kb) was used. Preliminary screening at the conventional *p* < 5 × 10^−8^ threshold indicated that too few independent variants would remain after clumping and harmonization to support a stable multi‐instrument analysis across both OA datasets; therefore, the suggestive‐threshold estimates were not considered confirmed by a strict‐threshold analysis. Instrument strength was evaluated using the F‐statistic, with F > 10 used as a conventional indicator of reduced weak‐instrument risk (Freeman et al. 2013). For this binary medication‐use phenotype, a reliable total liability‐scale *R* ^2^ could not be derived from the available harmonized summary output because compatible allele‐frequency and effect‐scale information was not consistently available for all retained SNPs. Accordingly, no quantified total proportion of exposure variance explained by the instrument set is reported.

$$F=\frac{R^{2}}{1-R^{2}}\times \frac{N-K-1}{K}$$

For individual instruments, *R* ^2^ may be approximated from effect estimates, standard errors, sample size, and allele frequency when these quantities are available on a compatible scale; the corresponding F‐statistic can then be derived from *R* ^2^, sample size, and the number of instruments. Because the required inputs were not consistently available for all retained SNPs in this binary medication‐use dataset, this methodological note is provided to clarify why a reliable total *R* ^2^ was not reported.

For individual instruments, *R* ^2^ may be approximated from effect estimates, standard errors, sample size, and allele frequency when these quantities are available on a compatible scale; the corresponding F‐statistic can then be derived from *R* ^2^, sample size, and the number of instruments. Because the required inputs were not consistently available for all retained SNPs in the binary medication‐use dataset, a reliable total *R* ^2^ was not reported.

### 2.3 Mendelian Randomization (MR) Analysis

This study employed the “TwoSampleMR” package in R software (version 4.3.3) for MR analysis. The results of the inverse‐variance weighted (IVW) method are used as the main basis, supplemented by MR‐Egger regression, weighted median (WM), simple mode, and weighted mode methods as complementary approaches to evaluate the association between genetic liability to insulin medication use and OA (Burgess and Thompson 2017; Bowden et al. 2016; Kennedy et al. 2020).

### 2.4 Sensitivity Analysis Plan

This study meticulously designed a sensitivity analysis strategy to ensure the accuracy and reliability of the results. Firstly, the MR‐Egger test was employed to rigorously assess potential horizontal pleiotropy (Liu et al. 2019). Specifically, if the intercept *p*‐value obtained from the test exceeds the threshold of 0.05, it can be inferred that there is no statistically significant horizontal pleiotropy, thereby enhancing the credibility of the analysis results (Bowden et al. 2015). To further detect and identify potential outliers that may contribute to pleiotropic bias, we introduced the MR‐PRESSO method. This method can precisely locate and analyze outliers, aiding us in gaining a deeper understanding of the data and reducing the impact of potential biases. Additionally, we applied Cochran's *Q* test to evaluate the heterogeneity of the instrumental variables (Bowden et al. 2019). When the *p*‐value is > 0.05, it is assumed that there is no significant heterogeneity among the instrumental variables, which helps to ensure the stability and consistency of the analysis results. Furthermore, we adopted the Leave‐One‐Out (LOO) method to comprehensively examine the robustness of the data. LOO analysis systematically excludes each data point one by one and recalculates based on the remaining data to test the stability and reliability of the analysis results (Deng et al. 2023).

### 2.5 Functional Annotation and Pathway Interpretation of Insulin‐Associated Genetic Variants

The 56 instrumental variables corresponded to genetic variants (SNPs) retained after harmonization with two OA outcome datasets. For biological interpretation, duplicated variants across datasets were removed, resulting in 29 unique insulin‐associated genetic variants for functional annotation. Standardized annotation was performed with Ensembl Variant Effect Predictor (VEP), ANNOVAR, FUMA, and Open Targets Genetics (https://genetics.opentargets.org/) (McLaren et al. 2016; Wang et al. 2010; Watanabe et al. 2017). Variant‐to‐gene mapping additionally incorporated expression quantitative trait locus (eQTL) and protein QTL (pQTL) evidence aggregated from these GWAS‐based resources where available. Each rsID was mapped to a genomic context and assigned a Sequence Ontology consequence where available, including missense, splice‐region, intronic, HLA‐region, and non‐coding regulatory/intergenic categories. Coding and splice‐region variants were further described using HGVS‐style transcript or protein annotations when transcript‐level information was available, whereas non‐coding and intergenic variants were classified by regulatory context and nearby or functionally linked genes/loci. The representative SNPs were mapped to nearby or functionally relevant genes/loci based on variant‐to‐gene annotation, genomic location, and reported biological relevance. The mapped genes/loci were then classified into functional categories, including regulatory/intergenic loci, immune‐inflammatory or HLA‐related loci, insulin biology/glucose metabolism‐related loci, transcription/chromatin regulation, and cellular signaling.

For gene‐based biological interpretation, Gene Ontology (GO) biological process and KEGG pathway analyses were performed using genes confidently mapped from insulin‐associated genetic variants. Enrichment was conducted using g:Profiler (organism: *Homo sapiens* ; sources: GO:BP and KEGG; g:Profiler version e114_eg62_p19_27110d83), with Benjamini‐Hochberg false discovery rate (FDR) correction applied to enrichment *p*‐values (Kolberg et al. 2023). A minimum mapped‐gene set size of 2 was required for reporting enriched terms, and FDR‐adjusted *p* < 0.05 was considered statistically significant. The analysis focused on biological processes and pathways related to insulin secretion, glucose homeostasis, immune regulation, inflammatory response, cytokine signaling, transcriptional regulation, PI3K‐Akt signaling, NF‐κB signaling, diabetes‐related pathways, and extracellular matrix remodeling. Bubble plots (Figure 7C,D) were generated to visualize the number of mapped genes and enrichment significance for each biological process or pathway, with bubble size reflecting gene count and color indicating FDR‐adjusted significance. The complete enrichment statistics, including term identifiers, mapped genes, raw *p*‐values where available, and FDR‐adjusted *p*‐values, are provided in Table S9.

### 2.6 Cell Culture and Treatment Strategies

The immortalized human chondrocyte cell line TC28a2 was used as an exploratory model. Cells were cultured in high‐glucose DMEM (25 mM glucose) containing 10% FBS at 37°C and 5% CO_2_. An initial concentration series included 10, 100, and 500 nM insulin for 6 h, based on the cited in vitro protocol (Ribeiro et al. 2016); the principal molecular analyses focused on 500 nM. This concentration is markedly higher than physiological fasting insulin and was used to examine early cellular responses rather than to model a clinically representative exposure. The study did not establish a complete dose–response relationship or compare multiple treatment durations.

#### 2.6.1 Safranin O Staining

Chondrocytes were washed with PBS 3 times after discarding the medium, 1 mL of safranin O dye was added to each well, and the cells were stained for 30–60 s, quickly dehydrated with absolute ethanol, and then observed and photographed as soon as possible. All the images were captured and then analyzed using a Nikon NIS Elements BR light microscope (Nikon, Tokyo, Japan). The staining intensity was determined by measuring the integrated optical density (IOD) in 10 different fields for each sample.

#### 2.6.2 Total RNA Extraction, Reverse Transcription, and RT‐qPCR Detection Methods

This study employed the TRIzol reagent to efficiently and non‐destructively extract high‐quality total RNA from TC28a2 cells. Subsequently, using the one‐stop reverse transcription quantitative polymerase‐chain‐reaction (RT‐qPCR) technology, complementary DNA (cDNA) was directly synthesized, significantly enhancing the convenience and accuracy of the experiment. To ensure the precision of the relative expression levels of mRNA, we strictly selected glyceraldehyde‐3‐phosphate dehydrogenase (GAPDH) as the internal reference gene for normalization, in order to eliminate experimental errors and reveal true biological differences. This normalization step is crucial for ensuring data reliability and comparability. Additionally, all primer sequences in this study were carefully designed and are detailed in Table S1, thereby enhancing the reproducibility and transparency of the experiment.

#### 2.6.3 Western Blotting (WB) Testing Method

This experiment utilized Western Blotting technology to detect specific proteins. Initially, tissue samples were lysed for 25 min in RIPA lysis buffer containing 1 mM PMSF under ice bath conditions to ensure sufficient cell disruption and protein release. Subsequently, the samples were centrifuged at 12,000 rpm at 4°C, and the supernatant was collected as the protein sample. To accurately quantify the proteins, a BCA protein quantification kit (P0010S, Beyotime Biotechnology Co. Ltd., Shanghai) was used for determination. Following this, proteins were separated using SDS‐PAGE technology and transferred onto high‐quality PVDF membranes (EMD Millipore Corporation, USA) (Zhang, Liu, et al. 2023). MMP13 (1:1000, Abcam, ab315267), MMP9 (1:2000, Abcam, ab283575), NRF2 (1:2000; Abcam, ab62352), GAPDH (1:10000; Abcam, ab8245) were used for incubation. Ultimately, ImageJ image analysis software was employed to precisely analyze the density of protein bands, thereby obtaining accurate protein expression data.

#### 2.6.4 Detection of Inflammatory Factor Levels Using ELISA Method

This investigation utilized the ELISA technique to measure inflammatory cytokines in TC28a2 cells. Initially, TC28a2 cells were plated at a concentration of 4 × 104 cells per compartment in a 24‐compartment culture dish. Afterward, varying concentrations of insulin (10, 100, or 500 nM) were administered to the cells. The concentrations of IL‐1β and IL‐6 were accurately determined using specific ELISA kits in accordance with the manufacturer's detailed guidelines.

#### 2.6.5 Statistical Analysis Methods

This study employed three professional software programs, namely SPSS 19, Image J, and Prism 9.3, for data analysis and processing. All quantitative data were accurately presented in the form of mean ± standard error of the mean (SEM). To rigorously evaluate the differences between the control group and the treatment group, we opted for the classic two‐tailed Student's *t*‐test. When the study involved comparisons between more than two groups, we employed one‐way analysis of variance (ANOVA) to comprehensively investigate the differences among the groups, supplemented by Tukey's post hoc test for further multiple comparisons. Additionally, leveraging the powerful capabilities of Prism 9.3 software, we calculated the Pearson correlation coefficient (*r*) between two variables (Kong et al. 2024). In this study, we strictly adhered to statistical principles, setting a *p*‐value < 0.05 as the threshold for determining statistically significant differences. This criterion ensures the scientific validity and reliability of our research conclusions.

## 3 Results

### 3.1 Genetic Liability to Insulin Medication Use Is Associated With Increased OA Risk

**FIGURE 2:**
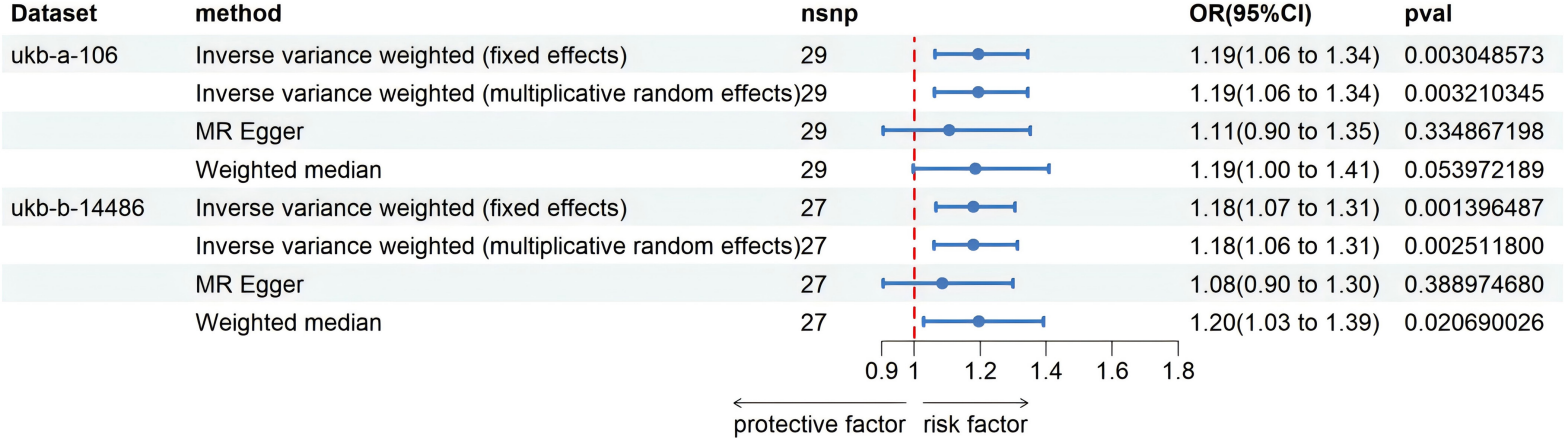
Overall MR forest plot showing the association between genetic liability to insulin medication use and osteoarthritis susceptibility. Each row represents an MR method. OR, odds ratio; CI, confidence interval. See Table S7 for additional abbreviations.

**FIGURE 3:**
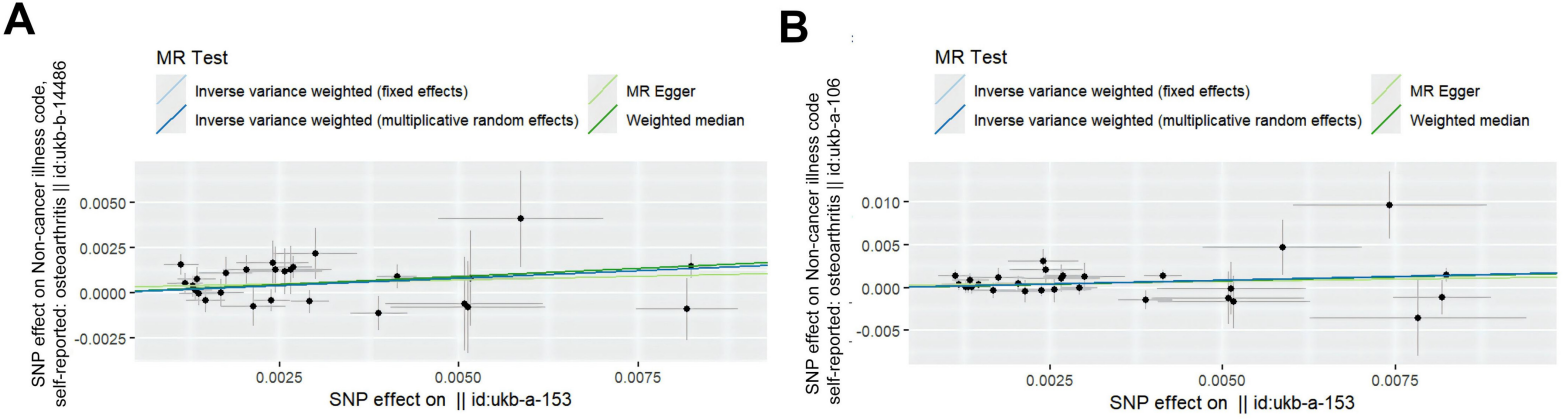
(A, B) MR scatter plots showing the association between genetic liability to insulin medication use and osteoarthritis susceptibility. Each point represents an instrumental genetic variant. The x‐axis shows the genetic variant effect on insulin medication use, and the y‐axis shows the genetic variant effect on osteoarthritis risk. Lines represent causal estimates from inverse‐variance weighted, MR‐Egger, weighted median, and weighted mode methods. Abbreviations: see Table S7.

**FIGURE 4:**
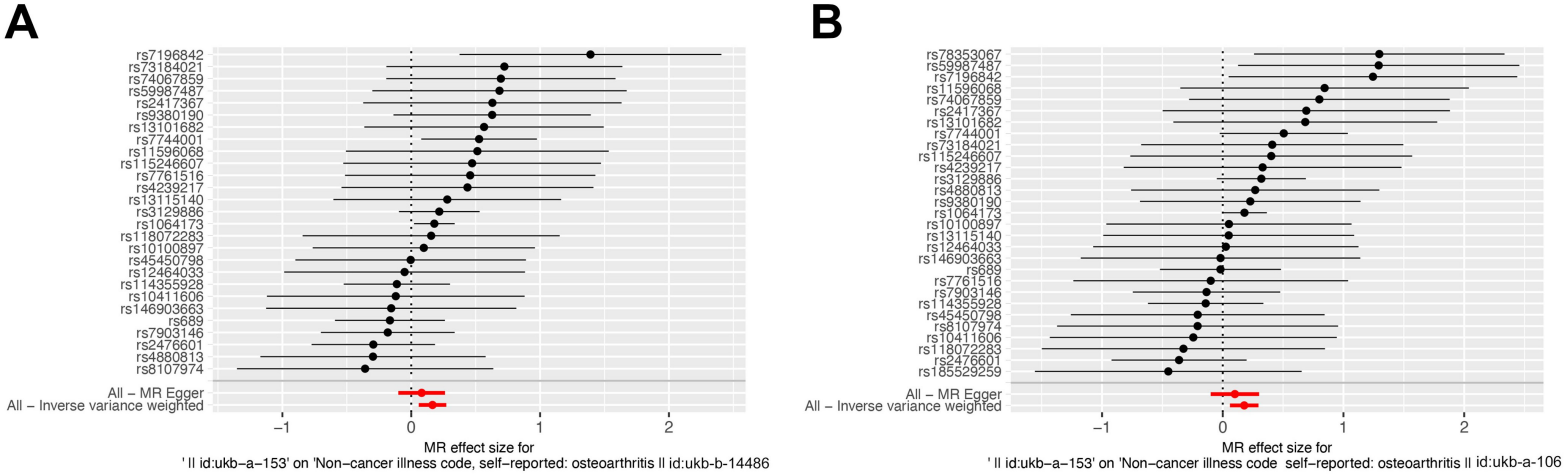
(A, B) Single‐SNP forest plot showing the effect of each individual insulin therapy‐associated genetic variant on osteoarthritis susceptibility. Each row represents an individual instrumental variable. Results are shown for the ukb‐b‐14486 (A) and ukb‐a‐106 (B) OA datasets. See Table S7 for abbreviations.

**FIGURE 5:**
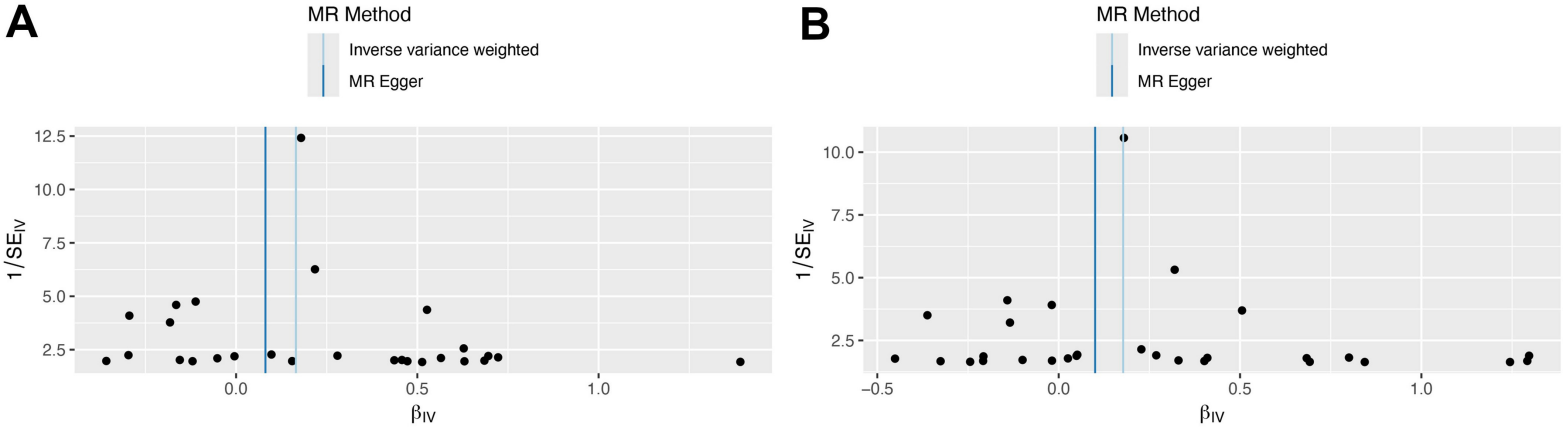
(A, B) Funnel plots assessing the symmetry of genetic variant effects for genetic liability to insulin medication use and osteoarthritis susceptibility. Abbreviations: see Table S7.

After a rigorous screening process, 56 insulin‐associated germline variants were identified as IVs, including 29 in the ukb‐a‐106 dataset and 27 in the ukb‐b‐14,486 dataset (Table S3). IVW analysis revealed a significant positive causal relationship between exogenous insulin and OA susceptibility in both datasets (ukb‐a‐106: OR = 1.1946, 95% CI: 1.0620–1.3436, *p* = 0.0030; ukb‐b‐14,486: OR = 1.1795, 95% CI: 1.0659–1.3052, *p* = 0.0014), further confirming that exogenous insulin is a risk factor for the development of OA (Figure 2, Table S3). Effect directions from the MR‐Egger and weighted median methods were consistent with the IVW estimates in both datasets (Table S3). Scatter plots (Figure 3), single‐SNP forest plots (Figure 4), and funnel plots (Figure 5) further supported a consistent, symmetric positive association between exogenous insulin and OA risk.

### 3.2 Sensitivity Analysis

**FIGURE 6:**
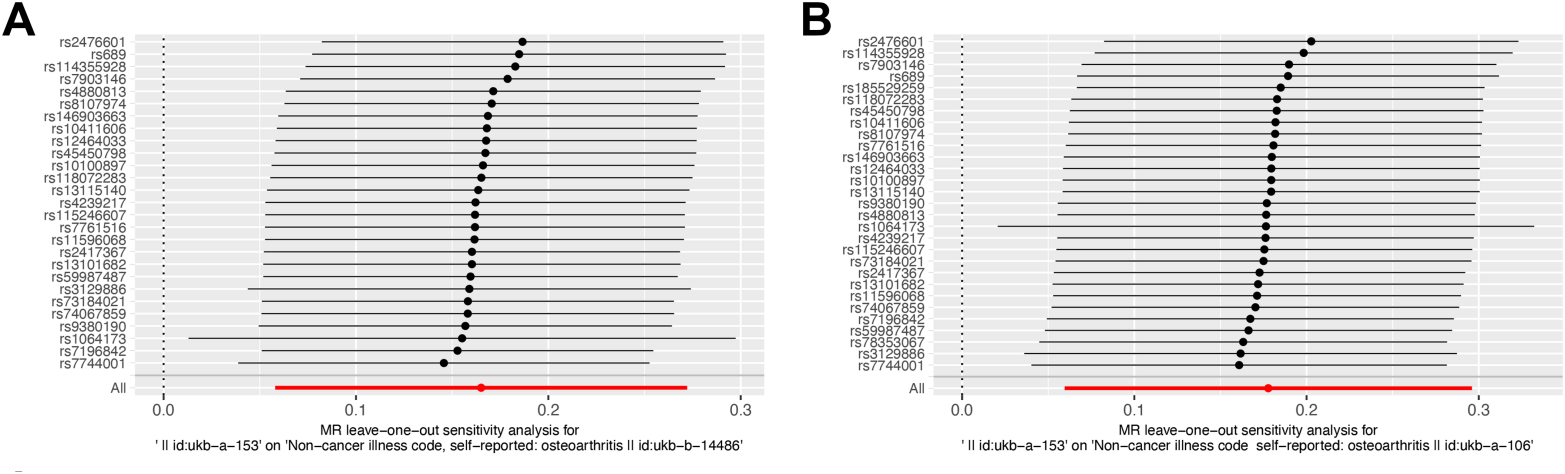
(A, B) Leave‐one‐out sensitivity analysis of the genetic variant‐based association between genetic liability to insulin medication use and osteoarthritis susceptibility. The analysis confirmed that no single genetic variant substantially drove the overall effect estimates. Abbreviations: see Table S7.

Sensitivity analyses were performed to evaluate the stability of the MR estimates. Cochran's *Q* tests showed no statistically significant heterogeneity (ukb‐a‐106: IVW *Q* = 28.304, *p* = 0.448; ukb‐b‐14,486: IVW *Q* = 29.070, *p* = 0.308) (Table S4). MR‐Egger intercepts did not indicate statistically significant directional horizontal pleiotropy (ukb‐a‐106: intercept = 0.000291, *p* = 0.357; ukb‐b‐14486: intercept = 0.000312, *p* = 0.271) (Table S5). MR‐PRESSO global tests were also non‐significant (ukb‐a‐106: RSSobs = 29.747, *p* = 0.484; ukb‐b‐14486: RSSobs = 30.821, *p* = 0.375) (Table S6), and leave‐one‐out analyses did not identify a single SNP that dominated the overall estimate (Figure 6). These statistical tests do not exclude indication‐related genetic liability, correlated pleiotropy, or alternative biological pathways arising from diabetes‐, immune‐, or inflammation‐related loci.

### 3.3 Functional Annotation of Insulin‐Associated Genetic Variants Highlights Metabolic and Immune‐Inflammatory Themes

**FIGURE 7:**
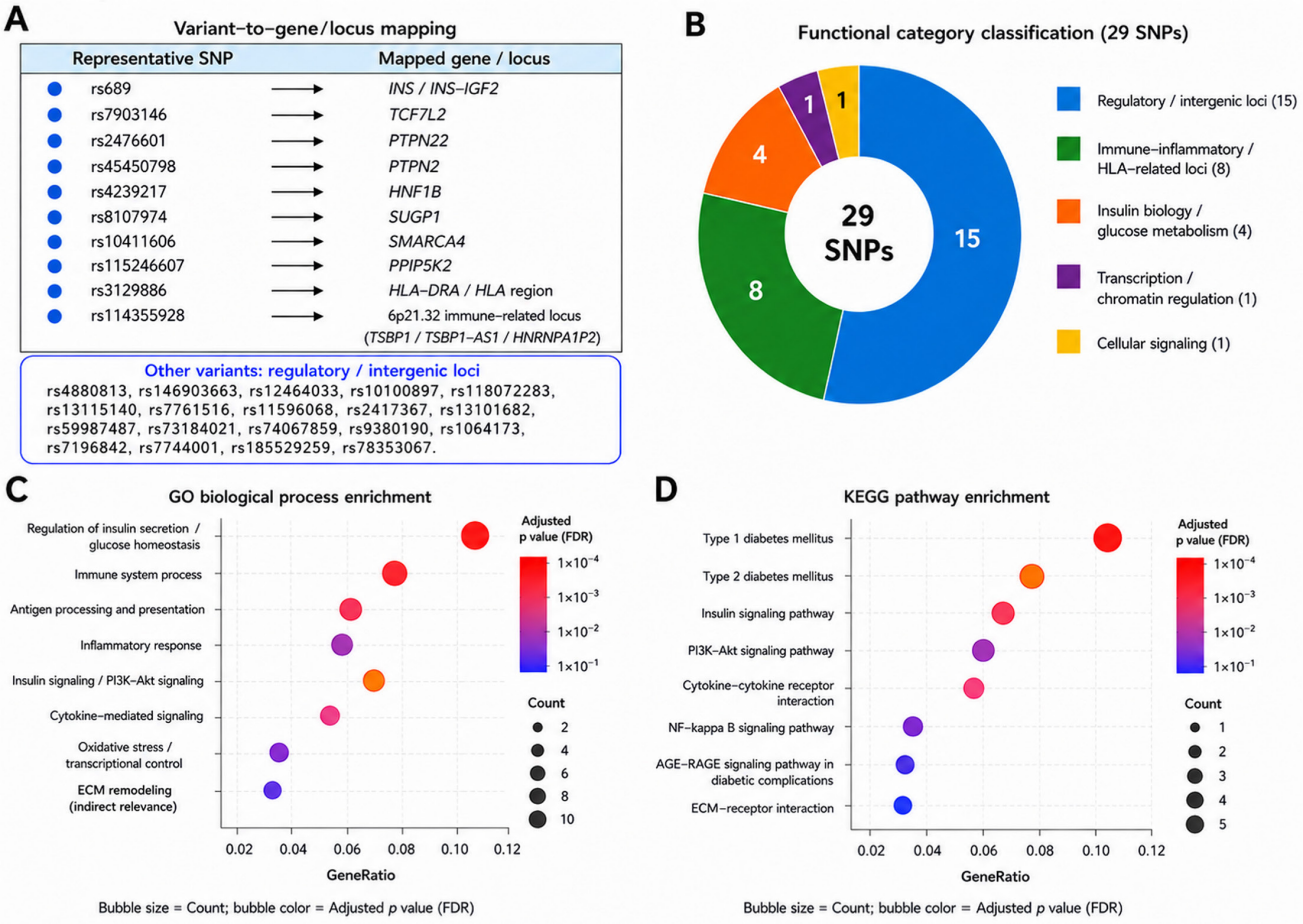
Functional annotation and classification of insulin‐associated genetic variants used as instrumental variables. (A) Representative variant‐to‐gene/locus mapping of insulin‐associated genetic variants. (B) Pie chart showing proportion of variant functional categories. (C) GO biological process enrichment bubble plot of the seven genes mapped from insulin‐associated genetic variants, showing all terms reaching FDR < 0.05. (D) KEGG pathway enrichment bubble plot of the same seven mapped genes. In (C, D), bubble size indicates the number of mapped genes annotated to the term and bubble colour indicates the FDR‐adjusted *p* value; GeneRatio is the number of mapped genes in the term divided by the seven input genes. In (D), terms marked with † are exploratory single‐gene‐mapped pathways that did not reach FDR < 0.05 and are shown for biological context only. Full enrichment output is provided in Table S9. FDR, false discovery rate; GO, Gene Ontology; KEGG, Kyoto Encyclopedia of Genes and Genomes.

To further investigate the biological meaning of the insulin‐associated genetic instruments, we performed functional annotation and classification of the SNPs used in the MR analysis. Variant annotation was carried out using Ensembl VEP, ANNOVAR, FUMA, and Open Targets Genetics, and 7 genes (INS, TCF7L2, HNF1B, PTPN22, PTPN2, HLA‐DRA, SMARCA4) were confidently mapped from the 29 unique insulin‐associated genetic variants (Table S8; Figure 7A).

Functional category classification showed that the largest proportion of SNPs belonged to regulatory or intergenic loci. Of the 29 unique SNPs, 15 were classified as regulatory/intergenic loci, 8 as immune‐inflammatory/HLA‐related loci, 4 as insulin‐biology/glucose‐metabolism loci, 1 as a transcription/chromatin‐regulation locus, and 1 as a cellular‐signaling locus (Figure 7B; Table S8).

Gene Ontology biological process enrichment analysis of the seven mapped genes identified FDR‐significant terms spanning both metabolic and immune‐inflammatory processes. Metabolic terms included glucose homeostasis (GO: 0042593; FDR = 1.28 × 10^−3^; mapped genes: INS, TCF7L2, SMARCA4, PTPN2), regulation of gluconeogenesis (GO: 0006111; FDR = 1.28 × 10^−3^; INS, TCF7L2, PTPN2), and insulin secretion (GO: 0030073; FDR = 2.08 × 10^−2^; TCF7L2, HNF1B). Immune‐related terms included T cell activation (GO: 0042110; FDR = 1.28 × 10^−3^; PTPN22, INS, SMARCA4, PTPN2, HLA‐DRA), lymphocyte activation (GO: 0046649; FDR = 2.00 × 10^−3^; PTPN22, INS, SMARCA4, PTPN2, HLA‐DRA), and positive regulation of cytokine production (GO: 0001819; FDR = 4.90 × 10^−2^; PTPN22, INS) (Figure 7C; Table S9). These GO terms reflect both the metabolic/glucose‐regulatory and immune‐inflammatory functions of the mapped genes.

Exploratory KEGG pathway enrichment analysis based on the seven mapped genes identified two pathways that reached FDR < 0.05: maturity onset diabetes of the young (KEGG:04950; FDR = 1.44 × 10^−2^; mapped genes: INS, HNF1B) and Type I diabetes mellitus (KEGG:04940; FDR = 1.81 × 10^−2^; mapped genes: INS, HLA‐DRA) (Figure 7D; Table S9). It should be noted that the small gene set (*n* = 7) limits the statistical power and generalizability of these pathway‐level interpretations. Additional single‐gene‐mapped KEGG terms (insulin secretion, Type II diabetes mellitus, insulin signaling, and PI3K‐Akt signaling; each supported only by INS) did not reach FDR significance and are presented in Table S9 as exploratory context.

### 3.4 Insulin Is Associated With Proteoglycan Reduction and Upregulation of Inflammatory Factors, Accompanied by NRF2 Downregulation

**FIGURE 8:**
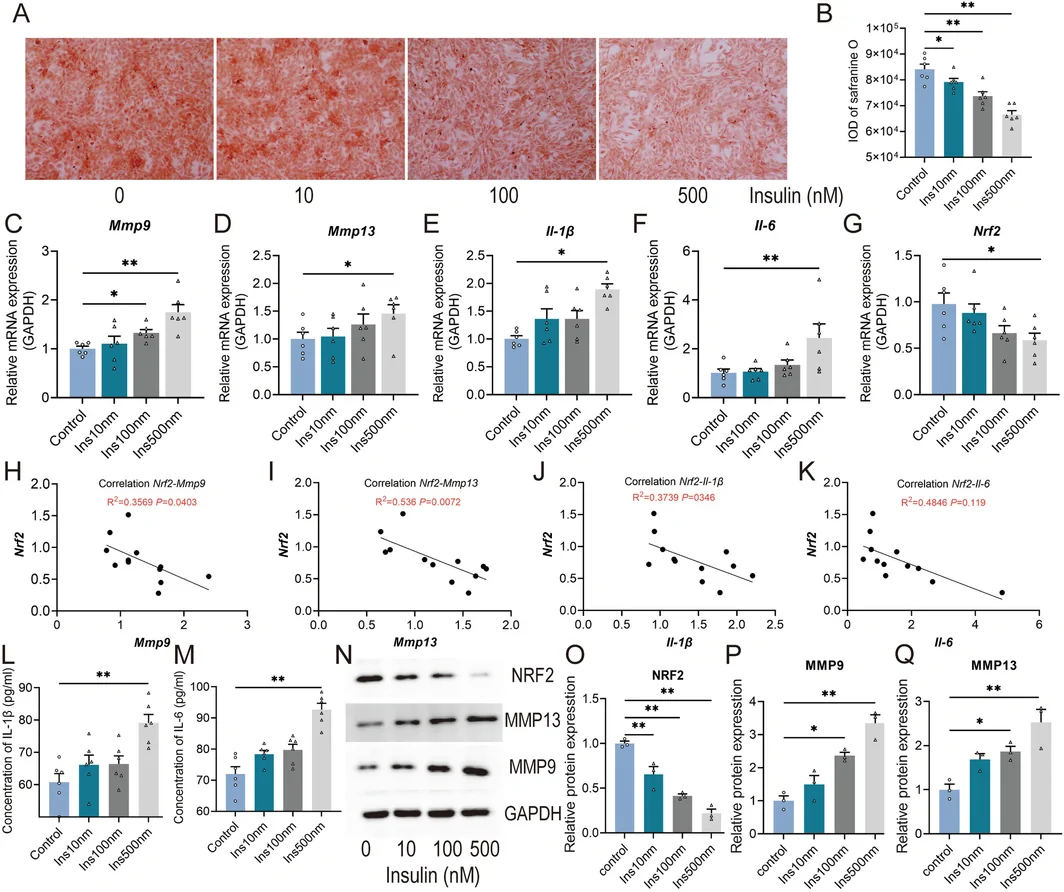
Insulin is associated with proteoglycan reduction and upregulation of inflammatory factors, accompanied by NRF2 downregulation, in TC28a2 chondrocytes. Cells were treated with 0 (control), 10, 100, or 500 nM insulin for 6 h under high‐glucose (25 mM) conditions. (A) Representative Safranin O staining images. (B) Integrated optical density (IOD) of Safranin O staining (*n* = 6). (C–G) Relative mRNA expression of MMP9 (C), MMP13 (D), IL‐1β (E), IL‐6 (F), and NRF2 (G), normalised to GAPDH (*n* = 6). (H–K) Correlation between NRF2 and MMP9 (H), MMP13 (I), IL‐1β (J), and IL‐6 (K) mRNA levels; each point represents an individual sample, with *R* ^2^ and *p* values indicated (*n* = 6). (L, M) Concentrations of IL‐1β (L) and IL‐6 (M) in culture supernatants determined by ELISA (*n* = 6). (N) Representative western blots of NRF2, MMP13, MMP9, and GAPDH. (O–Q) Quantification of NRF2 (O), MMP9 (P), and MMP13 (Q) protein levels normalised to GAPDH (*n* = 3). Data are presented as mean ± SEM. *p < 0.05, **p < 0.01 versus the 0 nM control group.

As a separate exploratory cellular experiment, TC28a2 cells were exposed to 500 nM insulin for 6 h under high‐glucose (25 mM) conditions (Ribeiro et al. 2016). Safranin O staining showed reduced proteoglycan staining, while RT‐qPCR, western blotting, and ELISA indicated increases in selected matrix‐degrading and inflammatory markers together with reduced NRF2 protein expression (Figure 8). The mRNA levels of the cartilage matrix synthesis genes COL2A1 and ACAN and of TNF‐α were measured under the same conditions and are shown in Figure S1. Full uncropped western blot images for GAPDH, MMP9, MMP13, and NRF2 are provided in Figures S2–S5. Because the experiment used an immortalized cell line, a supraphysiological insulin concentration, and high‐glucose medium, these observations cannot validate the MR association or establish that NRF2 downregulation mediates the cellular phenotype.

## 4 Discussion

OA is a common degenerative joint disorder with a substantial and increasing public‐health burden (Abramoff and Caldera 2020; Hawker 2019). In this study, MR analysis suggested an association between genetic liability to insulin medication use and OA susceptibility in two OA datasets. The estimates were directionally supported by complementary MR methods, and statistical sensitivity tests did not detect significant heterogeneity or directional horizontal pleiotropy. Nevertheless, the exposure represents self‐reported medication use rather than a direct insulin‐treatment contrast. Because insulin use is determined by diabetes diagnosis, severity, and treatment indication, and because many instruments map to diabetes‐related loci, the present design cannot distinguish a treatment‐specific insulin effect from indication‐related genetic liability, correlated pleiotropy, or other pathways related to the underlying disease.

Functional annotation provided biological context for the selected insulin medication‐use instruments. The mapped genes were linked to glucose metabolism, insulin secretion, immune regulation, and transcriptional control—processes potentially relevant to joint tissue homeostasis (Askari et al. 2017; Courties 2024). Several instruments are located in or near highly pleiotropic loci, including PTPN22, PTPN2, the HLA region, and TCF7L2, which have established roles in diabetes susceptibility, immune regulation, chronic inflammatory conditions, or other metabolic traits. These locus‐level annotations should not be interpreted as demonstrating an insulin‐specific pathway to OA. Although MR‐Egger and MR‐PRESSO did not identify statistically significant directional horizontal pleiotropy, correlated and biological pleiotropy operating through pathways independent of insulin medication use cannot be excluded.

The GO and KEGG enrichment analyses provided an exploratory bridge between the genetic findings and the cellular experiments. Of the 29 unique insulin‐associated genetic variants, only 7 genes (INS, TCF7L2, HNF1B, PTPN22, PTPN2, HLA‐DRA, SMARCA4) could be confidently mapped for enrichment analysis. With this small gene set, two KEGG pathways (maturity onset diabetes of the young and Type I diabetes mellitus) reached FDR < 0.05, while the enriched GO biological processes spanned both metabolic (glucose homeostasis, regulation of gluconeogenesis, insulin secretion) and immune‐inflammatory (T cell activation, lymphocyte activation, positive regulation of cytokine production) themes. Several biologically plausible pathways, including PI3K‐Akt signaling, insulin signaling, and Type II diabetes mellitus, were each supported by only a single gene (INS) and are therefore presented as exploratory context in Table S9 rather than as statistically significant enrichment results. We explicitly acknowledge that the small gene set (*n* = 7) limits the statistical power and generalizability of these pathway‐level interpretations, and these findings should be considered hypothesis‐generating rather than definitive.

Inflammation is categorized into sterile and bacterial types, and OA is a condition wherein the synovium, cartilage, and subchondral bone generate inflammatory agents, ultimately resulting in cartilage deterioration (Hodgkinson et al. 2022; Cai et al. 2015). Our in vitro experiments demonstrated that insulin treatment was associated with reduced NRF2 protein expression and concurrently increased MMP‐9 and MMP‐13 expression, accompanied by elevated levels of inflammatory cytokines (IL‐6, IL‐1β, and TNF‐α) in TC28a2 chondrocytes. These findings are consistent with the known role of NRF2 as a key transcriptional regulator of antioxidant and anti‐inflammatory genes (Sajadimajd and Khazaei 2017; He et al. 2020), and suggest a potential association between NRF2 suppression and the catabolic response observed in insulin‐treated chondrocytes (Wang and He 2022; Zhang, Jin, et al. 2023). However, it is important to emphasize that the current experimental evidence is correlational: we have not demonstrated through NRF2 gain‐ or loss‐of‐function experiments that NRF2 downregulation is causally responsible for the observed MMP upregulation or proteoglycan loss. The causal role of NRF2 in the insulin‐OA axis therefore awaits functional validation through NRF2 overexpression and silencing studies.

Notably, the data in Figure S1 reveal an asymmetric pattern of gene regulation in insulin‐treated TC28a2 chondrocytes: matrix‐degrading enzymes, including MMP‐9 and MMP‐13, were significantly upregulated, whereas cartilage anabolic genes such as COL2A1 and ACAN showed no statistically significant changes in expression. This asymmetry suggests that the catabolic effects of insulin on cartilage matrix metabolism are more pronounced than any potential anabolic effects under the experimental conditions tested. Matrix metalloproteinases are likewise a therapeutic focus in inflammatory arthritis, where agents suppressing MMP‐2 and MMP‐9 attenuate joint damage, and where biologic strategies developed for rheumatoid arthritis have been considered relevant to OA (Abramson and Yazici 2006; Liu et al. 2024).

In addition to direct effects on chondrocytes, insulin may influence OA progression through other avenues. Studies indicate that insulin can affect synovial cells and accelerate OA progression (Qiao et al. 2020). Insulin, as a type of medication, might result in hypoglycemia, which can cause falls or accidents, thus indirectly contributing to OA risk. Furthermore, insulin stimulates immune cells within adipose tissue to produce inflammatory signaling molecules, like IL‐6 and TNF‐α, potentially leading to a mild, widespread inflammatory condition that can instigate the advancement of OA (Nedunchezhiyan et al. 2022). These indirect mechanisms warrant further investigation in future studies. Other MR analyses have similarly implicated non‐articular pathways, such as the gut microbiota, in OA development (Yu et al. 2021).

Individuals utilizing insulin often experience weight gain, thereby increasing the strain on their joints and accelerating OA progression. Prior research has established that diabetes mellitus and OA are prevalent conditions frequently occurring together, particularly among those who are overweight or obese (Nedunchezhiyan et al. 2022). Consequently, individuals with osteoarthritis may necessitate regular insulin administration. Extensive studies affirm that both the occurrence and onset of diabetes escalate with advancing age (Umpierrez et al. 2024). This pattern is consistent across various countries, regions, and ethnic groups over time. In the United States, the prevalence of diabetes among those aged 60 and above can reach up to 20%, with elderly diabetes patients accounting for over 40% of the total diabetes population (Seow et al. 2024). Age is also a crucial factor in predicting OA, marked by a notable surge in cases of hand, hip, and knee OA (KOA) beyond the age of 50. Middle‐aged to elderly OA patients might need prolonged insulin therapy to manage blood glucose levels (Johnson and Hunter 2014; Allen and Golightly 2015; Neogi and Zhang 2013). Currently, as middle‐aged and elderly individuals age, their organ functions gradually decline, and their drug absorption, distribution, metabolism, and excretion differ significantly from younger adults. Therefore, in prescribing practices, clinicians should adopt a more comprehensive approach and conduct a thorough assessment of potential patient harm to guarantee medication efficacy and safety.

Several limitations should be emphasized. First, the exposure is self‐reported insulin medication use rather than circulating insulin or a randomized treatment contrast; therefore, the instruments may reflect diabetes liability, disease severity, and treatment indication, and the study cannot estimate an independent direct effect of insulin therapy. Second, MVMR was not performed in the current study. An adequately powered future MVMR analysis should adjust for diabetes liability, BMI, and glycemic traits while formally evaluating conditional instrument strength, collinearity, and potential sample overlap; without such analysis, the independent contribution of insulin medication use cannot be resolved (Hartley et al. 2022). Third, preliminary screening at *p* < 5 × 10^−8^ indicated that too few independent variants would remain for a stable multi‐instrument analysis, and a reliable total liability‐scale *R* ^2^ could not be derived from the available binary‐trait summary output. Fourth, non‐significant MR‐Egger and MR‐PRESSO tests do not exclude indication‐related genetic liability, correlated pleiotropy, or biological pleiotropy. Fifth, the enrichment analysis used only seven mapped genes and is exploratory. Sixth, the cellular experiment used immortalized TC28a2 cells, 500 nM insulin, and 25 mM glucose for 6 h without systematic viability testing, duration comparison, or NRF2 gain‐/loss‐of‐function analysis. These data provide preliminary cellular observations rather than functional validation of the MR association. Finally, the GWAS datasets were predominantly of European ancestry, limiting generalizability.

## 5 Conclusion

This study suggests an association between genetic liability to insulin medication use and increased osteoarthritis susceptibility. Sensitivity analyses did not detect statistically significant heterogeneity or directional horizontal pleiotropy; however, the medication‐use phenotype is strongly entangled with diabetes liability and treatment indication, and indication‐related genetic liability, correlated pleiotropy, and alternative biological pathways cannot be excluded. The analysis therefore cannot establish a treatment‐specific causal effect of exogenous insulin. Functional annotation highlighted metabolic and immune‐inflammatory themes, and a separate exploratory cellular experiment showed that insulin was associated with reduced proteoglycan content, increased expression of matrix‐degrading enzymes and inflammatory factors, and decreased NRF2 protein levels in chondrocytes. These cellular observations are hypothesis‐generating and do not validate the MR association or establish NRF2 downregulation as a causal mediator. The findings require confirmation in ancestry‐diverse and insulin‐specific designs, including appropriately powered multivariable MR, and in primary joint‐tissue models before clinical implications for patients receiving long‐term insulin therapy can be drawn.

## Author Contributions

**Yifan Liu:** validation, software. **Lingbo Rong:** conceptualization, methodology, writing – original draft. **Jiayong Zhu:** investigation, validation, methodology. **Tianyu Dai:** methodology, software. **Liaobin Chen:** funding acquisition, writing – review and editing, project administration. **Hui Wang:** funding acquisition, writing – review and editing, project administration.

## Funding

This work was supported by grants from the National Natural Science Foundation of China (No. U22A20362), and the National Key R&D Program of China (NO. 2020YFA0803900).

## Conflicts of Interest

The authors declare no conflicts of interest.

## Supporting information


**Table S1:** Oligonucleotide primers in quantitative real‐time PCR.
**Table S2:** Detailed genome‐wide association study (GWAS) data on exposure factors and outcomes.
**Table S3:** Statistical data of mendelian randomization analysis.
**Table S4:** Measures of heterogeneity using Cochran's *Q* test.
**Table S5:** Examination of horizontal pleiotropy effects using MR‐Egger regression tests.
**Table S6:** Examination of horizontal pleiotropy effects using MR‐PRESSO regression tests.
**Table S7:** List of abbreviations used in Figures 2–6.
**Table S8:** Functional annotation of 29 unique insulin‐associated genetic variants used as instrumental variables.
**Table S9:** Functional enrichment analysis of genes mapped from insulin‐associated genetic variants.
**Figure S1:** The mRNA expression of cartilage matrix synthesis genes and TNF‐α (A‐C) The mRNA expression of *COL2A1*, *ACAN*, *TNF‐α*; *n* = 6. Data are presented as Means ± SEM.
**Figure S2:** The full uncropped Gels and Blots image of GAPDH.
**Figure S3:** The full uncropped Gels and Blots image of MMP9.
**Figure S4:** The full uncropped Gels and Blots image of MMP13.
**Figure S5:** The full uncropped Gels and Blots image of NRF2.

## Data Availability

The data supporting the findings of this study are included in this article and its Supporting Information S1. Additional datasets generated or analyzed during the current study are available from the corresponding author upon reasonable request.
