# Supplementary material for: Integrative Multi‐Omics‐Informed Analysis Identifies Insulin Therapy–Associated Loci Implicated in Osteoarthritis Susceptibility
Source: Chem Biol Drug Des. 2026 Aug 26;108(2):e70384. doi: 10.1111/cbdd.70384 (PMC13510103; doi:10.1111/cbdd.70384)
Supplement: Supplementary file 1 [file CBDD-108-e70384-s001.docx]

**SUPPLEMENTARY MATERIAL**

Integrative Multi-Omics-Informed Analysis Identifies Insulin Therapy–Associated Loci Implicated in Osteoarthritis Susceptibility

Lingbo Rong ^1,#^, Jiayong Zhu ^2,#^, Yifan Liu ^3,#^, Tianyu Dai ^2^, Liaobin Chen ^2,3,*^, Hui Wang ^1,4,*^

1) **Supplementary Materials and Methods**
2) **Supplementary Tables 1-9**
3) **Supplementary Figures 1-5**

**Supplementary Materials and Methods**

**Experimental design**

The selected instruments were evaluated with reference to the three core MR assumptions: relevance to insulin medication use, independence from confounders, and absence of alternative pathways to OA. Because the exposure is a binary medication-use phenotype that overlaps genetically with diabetes liability and treatment indication, the independence and exclusion-restriction assumptions cannot be considered fully guaranteed. Statistical pleiotropy tests were therefore interpreted together with the biological functions of the selected loci.

**Instrument strength and threshold analysis**

The conventional genome-wide significance threshold (P < 5 × 10⁻⁸) yielded too few independent instruments after clumping and harmonization to support a stable multi-instrument MR analysis across both OA outcomes. For the binary medication-use phenotype, a reliable total liability-scale R² could not be derived because compatible allele-frequency and effect-scale information was not consistently available for all retained SNPs. Instrument strength was therefore assessed using available F-statistics, and the absence of a total R² estimate is acknowledged as a limitation.

**Cell Culture**

The cells were cultured in Dulbecco's modified Eagle's medium (DMEM) (Life Technologies, NY, USA) containing 10% fetal bovine serum (FBS, Life Technologies), and maintained in a constant temperature of 37°C and a 5% CO2 culture environment to ensure optimal conditions for cell growth.

**Statistical analysis methods**

Pearson's correlation coefficient (r) between two variables was calculated using Prism 9.3. The Pearson correlation coefficient ranges from -1 to 1, where -1 indicates a perfect negative correlation, 1 indicates a perfect positive correlation, and 0 indicates no correlation. The strength of the correlation was interpreted using guidelines such as the Cohen-sd effect size measure.

**Supplementary Tables**

**Supplementary Table 1.** Oligonucleotide primers in quantitative real-time PCR

| **Gene** | **Forward primer** | **Reverse primer** |
| --- | --- | --- |
| *MMP13* | GCACTTCCCACAGTGCCTAT | AGTTCTTCCCTTGATGGCCG |
| *MMP9* | TGCCATTCACGTCGTCCTTAT | CTGGGCTTAGATCATTCCTCA |
| *COL2A1* | GCTCCCAGAACATCACCTACC | CGATAACAGTCTTGCCCCAC |
| *ACAN* | CAGTGAGACGTCCGCCTATC | CGCCTCGCCTTCTTGAAATG |
| *NRF2* | CAACCCTTGTCACCATCTCAG | AGCGGCTTGAATGTTTGTCT |
| *IL-6* | ACCACCCCTGACCCAACC | AAGAAGGAATGCCCATTAACAA |
| *IL-1β* | CTGCCTGCCTTAGGGTAGTG | GGAGCGAATGACAGAGGGTTT |
| *TNF-α* | CACAGTGAAGTGCTGGCAAC | AGGAAGGCCTAAGGTCCACT |
| *GAPDH* | TCGGAGTCAACGGATTTGGT | TTCCCGTTCTCAGCCTTGAC |

**Supplementary Table 2.** Detailed genome-wide association study (GWAS) data on exposure factors and outcomes

|  | ukb-b-14,486 | ukb-a-106 | ukb-a-153 |
| --- | --- | --- | --- |
| Year | 2018 | 2017 | 2017 |
| Category | Binary | N/A | N/A |
| Sub category | N/A | N/A | N/A |
| Race | European | European | European |
| Gender | Males and Females | Males and Females | Males and Females |
| Number of Case Group | 38472 | 28257 | 19668 |
| Number of Control Group | 424461 | 308902 | 333840 |
| Sample Size | 462933 | 337159 | 337159 |
| Number of SNPs | 9851867 | 10894596 | 10894596 |

**Supplementary Table 3.** Statistical data of mendelian randomization analysis

| Dataset | method | nsnp | pval | or | or_lci95 | or_uci95 |
| --- | --- | --- | --- | --- | --- | --- |
| ukb-a-106 | Inverse variance weighted  (fixed effects) | 29 | 0.003048573 | 1.194558776 | 1.06201803 | 1.343640719 |
|  | Inverse variance weighted  (multiplicative random effects) | 29 | 0.003210345 | 1.194558776 | 1.061342106 | 1.344496427 |
|  | MR Egger | 29 | 0.334867198 | 1.105667742 | 0.904782177 | 1.351155213 |
|  | Weighted median | 29 | 0.053972189 | 1.185277615 | 0.997098706 | 1.408970862 |
| ukb-b-14486 | Inverse variance weighted  (fixed effects) | 27 | 0.001396487 | 1.179488561 | 1.065903233 | 1.305177826 |
|  | Inverse variance weighted (multiplicative random effects) | 27 | 0.0025118 | 1.179488561 | 1.059725858 | 1.312785995 |
|  | MR Egger | 27 | 0.38897468 | 1.084400795 | 0.904734461 | 1.299746098 |
|  | Weighted median | 27 | 0.020690026 | 1.195732775 | 1.027696937 | 1.391243681 |

**Supplementary Table 4.** Measures of heterogeneity using Cochran’s Q test

| Exposure | Outcome | Method | Q | Q_df | Q_pval |
| --- | --- | --- | --- | --- | --- |
| ukb-a-153 | ukb-a-106 | MR Egger | 27.41252917 | 27 | 0.441716538 |
| ukb-a-153 | ukb-a-106 | Inverse variance weighted | 28.30397402 | 28 | 0.448429774 |
| ukb-a-153 | ukb-b-14486 | MR Egger | 27.67089723 | 25 | 0.323224448 |
| ukb-a-153 | ukb-b-14486 | Inverse variance weighted | 29.07049904 | 26 | 0.307885968 |

**Supplementary Table 5.** Examination of horizontal pleiotropy effects using MR-Egger regression tests

| id.exposure | id.outcome | exposure | egger_intercept | se | pval |
| --- | --- | --- | --- | --- | --- |
| ukb-a-153 | ukb-a-106 | \|\| id:ukb-a-153 | 0.00029113 | 0.000310694 | 0.357048562 |
| ukb-a-153 | ukb-b-14486 | \|\| id:ukb-a-153 | 0.00031197 | 0.000277429 | 0.271481088 |

**Supplementary Table 6.** Examination of horizontal pleiotropy effects using MR-PRESSO regression tests

|  | id.exposure | id.outcome | RSSobs | pval |
| --- | --- | --- | --- | --- |
| presso2 | ukb-a-153 | ukb-b-14,486 | 30.82106 | 0.375 |
| presso2.1 | ukb-a-153 | ukb-a-106 | 29.74677 | 0.484 |

**Supplementary Table 7.** List of abbreviations used in Figures 2–6

| **Abbreviation** | **Full term** |
| --- | --- |
| MR | Mendelian randomization |
| SNP | Single nucleotide polymorphism |
| IV | Instrumental variable |
| IVs | Instrumental variables |
| IVW | Inverse variance weighted |
| MR-Egger | Mendelian randomization–Egger regression |
| WM | Weighted median |
| MR-PRESSO | Mendelian randomization pleiotropy residual sum and outlier |
| OR | Odds ratio |
| 95% CI | 95% confidence interval |
| SE | Standard error |
| LOO | Leave-one-out analysis |
| GWAS | Genome-wide association study |
| OA | Osteoarthritis |
| UKB | UK Biobank |
| ukb-a-153 | UK Biobank GWAS dataset for insulin medication use |
| ukb-a-106 | UK Biobank GWAS dataset for self-reported osteoarthritis |
| ukb-b-14486 | UK Biobank GWAS dataset for osteoarthritis (MRC-IEU) |
| *P* | *P*-value |

**Supplementary Table 8.** Functional annotation of 29 unique insulin-associated genetic variants used as instrumental variables

Variant annotation was performed using Ensembl Variant Effect Predictor (VEP), ANNOVAR, FUMA, and Open Targets Genetics. Variant consequences are reported using Sequence Ontology (SO) terms. HGVS annotations are provided where transcript-level evidence was available. Non-coding and intergenic genetic variants are classified by regulatory context and nearby or functionally linked genes/loci. Functional categories follow the classification described in the main text: 15 regulatory/intergenic loci, 8 immune-inflammatory or HLA-related loci, 4 insulin biology/glucose metabolism-related loci, 1 transcription/chromatin regulation, and 1 cellular signaling.

| SNP | Mapped gene/locus  (genomic location) | Consequence  (Sequence Ontology) | HGVS annotation | Functional category | Biological interpretation |
| --- | --- | --- | --- | --- | --- |
| rs689 | INS / INS-IGF2  (chr11:2,160,994) | splice_region_variant  (SO:0001630) | NC_000011.10: g.2182229_  2182230ins (INS splice-  region) | Insulin biology  / glucose metabolism | INS is directly involved in insulin production and secretion; this genetic variant provides a direct genetic link to insulin-related exposure. |
| rs7903146 | TCF7L2  (chr10:112,998,590) | intron_variant  (SO:0001627) | NC_000010.11: g.114758349  C>T (TCF7L2 intronic;  major T2D GWAS locus) | Insulin biology /glucose metabolism | TCF7L2 is a major type 2 diabetes-associated gene involved in glucose homeostasis and insulin secretion. |
| rs4239217 | HNF1B  (chr17:37,738,996) | intron_variant  (SO:0001627) | N/A (intronic/regulatory) | Insulin biology /glucose metabolism | HNF1B is a transcription factor involved in metabolic regulation and has been linked to diabetes-related genetic architecture. |
| rs8107974 | SUGP1  (chr19:19,277,691) | intron_variant  (SO:0001627) | N/A (intronic) | Insulin biology /glucose metabolism | SUGP1 encodes a splicing-related protein reported in metabolic trait GWAS contexts. |
| rs2476601 | PTPN22 / AP4B1-AS1  (chr1:113,834,946) | missense_variant  (SO:0001583) | NM_015967.7: c.1858C>T NP_057051.2: p. Arg620Trp | Immune-inflammatory/ HLA-related | PTPN22 is involved in immune-cell signaling and autoimmune susceptibility; this missense variant supports an immune-inflammatory component in insulin-related OA risk. |
| rs45450798 | PTPN2  (chr18:12,792,941) | intron_variant (SO:0001627) | N/A (intronic) | Immune-inflammatory / HLA-related | PTPN2 is associated with immune regulation and type 1 diabetes-related genetic susceptibility. |
| rs114355928 | TSBP1 / TSBP1-AS1  (chr6:32,298,104) | intron_variant (SO:0001627) | N/A (intronic; HLA region) | Immune-inflammatory / HLA-related | Located in the 6p21.32 immune-related HLA region, suggesting possible involvement of immune and inflammatory regulation. |
| rs3129886 | HLA-DRA / HLA class II  region  (chr6:32,400,000; 6p21.32) | HLA_region_variant (regulatory/non-coding) | N/A (HLA non-coding region) | Immune-inflammatory / HLA-related | Located in the HLA class II region near HLA-DRA; supports immune-inflammatory interpretation of the instrument set. |
| rs9380190 | HLA/MHC-related locus  (chr6:30,801,788) | regulatory_region_variant (SO:0001887; non-coding) | N/A (non-coding regulatory) | Immune-inflammatory / HLA-related | Classified as an immune-region regulatory genetic variant in the HLA/MHC locus. |
| rs1064173 | HLA/MHC-related locus  (chr6:32,659,703) | regulatory_region_variant (SO:0001887; non-coding) | N/A (non-coding regulatory) | Immune-inflammatory / HLA-related | Classified as an immune-region regulatory genetic variant in the HLA/MHC locus. |
| rs7744001 | HLA/MHC-related locus  (chr6:32,658,309) | regulatory_region_variant (SO:0001887; non-coding) | N/A (non-coding regulatory) | Immune-inflammatory / HLA-related | Classified as an immune-region regulatory genetic variant in the HLA/MHC locus. |
| rs7761516 | Intergenic/regulatory locus  (chr6:138,338,848) | intergenic_variant (SO:0001628; non-coding  regulatory) | N/A (non-coding intergenic) | Immune-inflammatory/ HLA-related | Classified as a non-coding regulatory locus near the HLA region, likely contributing to immune-related genetic background. |
| rs10411606 | SMARCA4  (chr19:11,062,453) | intron_variant (SO:0001627) | N/A (intronic) | Transcription /chromatin regulation | SMARCA4 participates in chromatin remodeling and transcriptional regulation, which may influence downstream metabolic or inflammatory responses. |
| rs115246607 | PPIP5K2  (chr5:103,126,186) | intron_variant (SO:0001627) | N/A (intronic) | Cellular signaling | PPIP5K2 is involved in inositol phosphate metabolism and cellular signaling. |
| rs4880813 | Regulatory/intergenic locus  (chr10:1,295,973) | intergenic_variant (SO:0001628; non-coding regulatory) | N/A (non-coding intergenic) | Regulatory / intergenic | Classified as a non-coding insulin-associated locus; included in variant-level functional interpretation but not used for gene-based enrichment. |
| rs146903663 | Regulatory/intergenic locus  (chr1:241,575,523) | intergenic_variant (SO:0001628; non-coding regulatory) | N/A (non-coding intergenic) | Regulatory / intergenic | Classified as a non-coding insulin-associated locus; may reflect regulatory variation rather than direct coding-gene disruption. |
| rs12464033 | Regulatory/intergenic locus  (chr2:203,931,371) | intergenic_variant (SO:0001628; non-coding regulatory) | N/A (non-coding intergenic) | Regulatory / intergenic | Classified as a non-coding insulin-associated locus and retained for variant-level interpretation. |
| rs10100897 | Regulatory/intergenic locus  (chr8:521,805) | intergenic_variant (SO:0001628; non-coding regulatory) | N/A (non-coding intergenic) | Regulatory / intergenic | Classified as a non-coding insulin-associated regulatory locus. |
| rs118072283 | Regulatory/intergenic locus  (chr10:78,966,660) | intergenic_variant (SO:0001628; non-coding regulatory) | N/A (non-coding intergenic) | Regulatory / intergenic | Classified as a non-coding insulin-associated regulatory locus. |
| rs13115140 | Regulatory/intergenic locus  (chr4:6,313,707) | intergenic_variant (SO:0001628; non-coding regulatory) | N/A (non-coding intergenic) | Regulatory / intergenic | Classified as a non-coding insulin-associated regulatory locus. |
| rs11596068 | Regulatory/intergenic locus  (chr10:60,127,233) | intergenic_variant (SO:0001628; non-coding regulatory) | N/A (non-coding intergenic) | Regulatory / intergenic | Classified as a non-coding insulin-associated regulatory locus. |
| rs2417367 | Regulatory/intergenic locus  (chr9:102,494,715) | intergenic_variant (SO:0001628; non-coding regulatory) | N/A (non-coding intergenic) | Regulatory / intergenic | Classified as a non-coding regulatory locus in the insulin-associated variant set. |
| rs13101682 | Regulatory/intergenic locus  (chr4:56,869,192) | intergenic_variant (SO:0001628; non-coding regulatory) | N/A (non-coding intergenic) | Regulatory / intergenic | Classified as a non-coding insulin-associated regulatory locus. |
| rs59987487 | Regulatory/intergenic locus  (chr1:147,417,389) | intergenic_variant (SO:0001628; non-coding regulatory) | N/A (non-coding intergenic) | Regulatory / intergenic | Classified as a non-coding insulin-associated regulatory locus. |
| rs73184021 | Regulatory/intergenic locus  (chr7:104,886,087) | intergenic_variant (SO:0001628; non-coding regulatory) | N/A (non-coding intergenic) | Regulatory / intergenic | Classified as a non-coding insulin-associated regulatory locus. |
| rs74067859 | Regulatory/intergenic locus  (chr12:21,909,964) | intergenic_variant (SO:0001628; non-coding regulatory) | N/A (non-coding intergenic) | Regulatory / intergenic | Classified as a non-coding insulin-associated regulatory locus. |
| rs7196842 | Regulatory/intergenic locus  (chr16:69,889,430) | intergenic_variant (SO:0001628; non-coding regulatory) | N/A (non-coding intergenic) | Regulatory / intergenic | Classified as a non-coding insulin-associated regulatory locus. |
| rs185529259 | Regulatory/intergenic locus  (chr2:156,050,872) | intergenic_variant (SO:0001628; non-coding regulatory) | N/A (non-coding intergenic) | Regulatory / intergenic | Classified as a non-coding insulin-associated regulatory locus. |
| rs78353067 | Regulatory/intergenic locus  (chr8:128,083,227) | intergenic_variant (SO:0001628; non-coding regulatory) | N/A (non-coding intergenic) | Regulatory / intergenic | Classified as a non-coding insulin-associated regulatory locus. |

Abbreviations: SO, Sequence Ontology; VEP, Ensembl Variant Effect Predictor; HGVS, Human Genome Variation Society; HLA, human leukocyte antigen; MHC, major histocompatibility complex; GWAS, genome-wide association study; T2D, type 2 diabetes; Functional category summary: 4 insulin biology/glucose metabolism, 8 immune-inflammatory/HLA-related, 1 transcription/chromatin regulation, 1 cellular signaling, 15 regulatory/intergenic (total = 29 genetic variants).

**Supplementary Table 9.** Functional enrichment analysis of genes mapped from insulin-associated genetic variants

Enrichment was performed using g: Profiler (organism: Homo sapiens; sources: GO: BP and KEGG; g:Profiler version e114_eg62_p19_27110d83), with Benjamini-Hochberg false discovery rate (FDR) correction applied to enrichment p-values. Terms with FDR-adjusted p < 0.05 were considered statistically significant. Input genes confidently mapped from insulin-associated genetic variants: INS, TCF7L2, HNF1B, PTPN22, PTPN2, HLA-DRA, SMARCA4 (n = 7). The analysis focused on biological processes and pathways related to insulin secretion, glucose homeostasis, immune regulation, inflammatory response, cytokine signaling, and diabetes-related pathways.

| **Database** | **Term ID** | **Pathway / biological process** | **Mapped genes (gene count)** | **Raw p-value** | **FDR-adjusted p-value** | **FDR < 0.05** | **Enrichment interpretation** |
| --- | --- | --- | --- | --- | --- | --- | --- |
| **FDR-Significant GO Biological Process Terms (FDR < 0.05)** | | | | | | | |
| GO: BP | GO:0042593 | Glucose homeostasis | INS, TCF7L2, SMARCA4, PTPN2 (4/7) | 6.87 × 10⁻⁴ | 1.28 × 10⁻³ | Yes | Supports metabolic and glucose-regulatory relevance of insulin-associated genetic variants. |
| GO: BP | GO:0006111 | Regulation of gluconeogenesis | INS, TCF7L2, PTPN2 (3/7) | 4.26 × 10⁻⁴ | 1.28 × 10⁻³ | Yes | Links mapped genes to glucose production and systemic metabolic regulation. |
| GO: BP | GO:0042110 | T cell activation | PTPN22, INS, SMARCA4, PTPN2, HLA-DRA (5/7) | 3.09 × 10⁻⁴ | 1.28 × 10⁻³ | Yes | Supports an immune-inflammatory component involving T cell activity relevant to OA pathogenesis. |
| GO: BP | GO:0046649 | Lymphocyte activation | PTPN22, INS, SMARCA4, PTPN2, HLA-DRA (5/7) | 1.62 × 10⁻³ | 2.00 × 10⁻³ | Yes | Highlights broader lymphocyte activation, consistent with immune-mediated OA mechanisms. |
| GO: BP | GO:0030073 | Insulin secretion | TCF7L2, HNF1B (2/7) | — | 2.08 × 10⁻² | Yes | Supports biological relevance to insulin secretion and insulin-related exposure. |
| GO: BP | GO:0001819 | Positive regulation of cytokine production | PTPN22, INS (2/7) | — | 4.90 × 10⁻² | Yes | Connects mapped genes to cytokine production relevant to inflammatory OA progression. |
| **FDR-Significant KEGG Pathway Terms (FDR < 0.05)** | | | | | | | |
| KEGG | KEGG:04950 | Maturity onset diabetes of the young | INS, HNF1B (2/7) | 6.44 × 10⁻³ | 1.44 × 10⁻² | Yes | FDR-significant diabetes-related pathway involving INS and HNF1B, consistent with the metabolic relevance of insulin-associated genetic variants. |
| KEGG | KEGG:04940 | Type I diabetes mellitus | INS, HLA-DRA (2/7) | 1.62 × 10⁻² | 1.81 × 10⁻² | Yes | FDR-significant immune/insulin-related diabetes pathway involving INS and HLA-DRA, supporting both metabolic and immune-inflammatory components. |
| **Exploratory KEGG Pathway-Level Signals † (FDR ≥ 0.05; single-gene mapping)** | | | | | | | |
| KEGG | KEGG:04911 | Insulin secretion | INS (1/7) † | — | 1.72 × 10⁻¹ | No | Exploratory insulin secretion pathway-level signal; not FDR-significant in the small mapped-gene set. Included for biological context. |
| KEGG | KEGG:04930 | Type II diabetes mellitus | INS (1/7) † | — | 1.72 × 10⁻¹ | No | Exploratory type II diabetes pathway-level signal; not FDR-significant. Included for biological context. |
| KEGG | KEGG:04910 | Insulin signaling pathway | INS (1/7) † | — | 1.73 × 10⁻¹ | No | Exploratory insulin signaling pathway-level signal; not FDR-significant. Included for biological context. |
| KEGG | KEGG:04151 | PI3K-Akt signaling pathway | INS (1/7) † | — | 2.71 × 10⁻¹ | No | Exploratory PI3K-Akt pathway-level signal; biologically consistent with in vitro findings showing insulin-mediated inflammatory and matrix-degrading effects in chondrocytes. Not FDR-significant. |

KEGG terms with single-gene mapping (INS only) are included in the Exploratory section for biological context and pathway-level interpretation. These terms do not meet the formal enrichment threshold (FDR ≥ 0.05) and should not be interpreted as statistically significant enrichment results. Abbreviations: GO: BP, Gene Ontology Biological Process; KEGG, Kyoto Encyclopedia of Genes and Genomes; FDR, false discovery rate (Benjamini-Hochberg correction). Raw p-values for GO:0042593, GO:0006111, GO:0042110, GO:0046649, KEGG:04950, and KEGG:04940 were obtained from g: Profiler (organism: Homo sapiens; g: Profiler version e114_eg62_p19_27110d83; accessed 2026-05-26).

**Supplementary Figures**

**Supplementary Figure 1. The mRNA expression of cartilage matrix synthesis genes and TNF-α**. (A-C) The mRNA expression of *COL2A1*, *ACAN*, *TNF-α*; n = 6. Data are presented as Means ± S.E.M.

**Supplementary Figure 2. The full uncropped Gels and Blots image of GAPDH**.

**Supplementary Figure 3. The full uncropped Gels and Blots image of MMP9**.

**Supplementary Figure 4. The full uncropped Gels and Blots image of MMP13**.

**Supplementary Figure 5. The full uncropped Gels and Blots image of NRF2**.
